# Long term unemployment, income, poverty, and social public expenditure, and their relationship with self-perceived health in Spain (2007–2011)

**DOI:** 10.1186/s12889-017-5004-2

**Published:** 2018-01-15

**Authors:** M. Puerto López del Amo González, Vivian Benítez, José J. Martín-Martín

**Affiliations:** Applied Economics Department, University of Granada, Facultad de Ciencias Económicas y Empresariales, Campus Universitario de Cartuja s/n 18011, Granada, Spain

**Keywords:** Long term unemployment, Self-reported health, Spain, Multilevel logistic longitudinal regression, Great recession, Income, Poverty, Social public expenditure, Social health determinants

## Abstract

**Background:**

There is scant research that simultaneously analyzes the joint effects of long-term unemployment, poverty and public expenditure policies on poorer self-perceived health during the financial crisis. The aim of the study is to analyze the joint relationship between long-term unemployment, social deprivation, and regional social public expenditure on one side, and self-perceived health in Spain (2007–2011) on the other.

**Methods:**

Longitudinal data were extracted from the Survey on Living Conditions, 2007–2010 and 2008–2011 (9105 individuals and 36,420 observations), which were then used to estimate several random group effects in the constant multilevel logistic longitudinal models (level 1: year; level 2: individual; level 3: region). The dependent variable was self-perceived health. Individual independent interest variables were long and very long term unemployment, available income, severe material deprivation and regional variables were per capita expenditure on essential public services and per capita health care expenditure.

**Results:**

All of the estimated models show a robust association between bad perceived health and the variables of interest. When compared to employed individuals, long term unemployment increases the odds of reporting bad health by 22% to 67%; very long-term unemployment (24 to 48 months) increases the odds by 54% to 132%. Family income reduces the odds of reporting bad health by 16% to 28% for each additional percentage point in income. Being a member of a household with severe material deprivation increases the odds of perceiving one’s health as bad by between 70% and 140%. Regionally, per capita expenditure on essential public services increases the odds of reporting good health, although the effect of this association was limited.

**Conclusions:**

Long and very long term unemployment, available income and poverty were associated to self-perceived bad health in Spain during the financial crisis. Regional expenditure on fundamental public services is also associated to poor self-perceived health, although in a more limited fashion. Results suggest the positive role in health of active employment and redistributing income policies.

## Background

The financial crisis, which started in 2008, has brought about a social crisis, which has worsened the health conditions of individuals throughout Europe [1, 2], with particularly severe effects in Southern countries [3, 4], due to the conjunction of recession and stark austerity policies [5, 6].

In Spain the crisis has caused a dramatic increase in unemployment and poverty, while social protection policies have weakened [7, 8]. Unemployment skyrocketed from 8.3% in 2007 to 28.1% in 2013, the last year of the crisis, whereas these deleterious effects were much more moderate in other European countries [9]. One of the main reasons for this sudden increase is the historical dualization of the Spanish labor market since the year 1984, when a binary divide was established between indefinite contracts, with high severance pay for laidoff workers, and temporary contracts with low severance compensation. Before the financial crisis, 88% of the 18.5 million yearly contracts were temporary. For the first few years of the crisis, the increase in unemployment affected mainly workers with the latter modality of contract, which went from 31.6% in 2007 to 21.9% in early 2013 [10]. In other words, the strong increase in unemployment was caused by the harsh dualization of the labor market and the asymmetrical impact of the financial crisis, and is probably not closely related to the health of workers. This labor dynamic is in agreement with current models used in institutions and in the theory of labor segmentation [11].

Between 2007 and 2011 long-term unemployment increased in Spain from 22% to 43% [12]. This increase, along with wage deflation, has caused an escalation of poverty and a drop in available income [13]. In the period 2007–2011, available income of Spanish households in constant 2011 Euros dropped from 26,773 to 22,146 [14] and severe material deprivation rose by 53% between 2009 and 2014, leaving 7% of the Spanish population in such a dire situation [15].

The existing literature does not agree on the definition of long-term unemployment. Brenner et al. (2016) or Romeu (2006) classified unemployment as long term when it lasts between one and two years, and as very long term when it lasts over two years [16, 17]. In other works, long term designates unemployment over one year in length [18, 19], over two years [20, 21], between one and three years [22] or over five years [23]. For the purposes of our study we will follow the definition of Brenner et al. (2016) [16].

This evidence at the international level regarding the effect of long-term unemployment on health shows contradictory conclusions. Some studies found a negative impact of long-term unemployment on health [16–18, 22, 23] whereas others found no such link [20, 24].

For instance, in 2013 Herbig et al. reviewed the existing literature to conclude that long-term unemployment increases mortality and the incidence of the most prevalent mental conditions [18]. Along the same lines, a recent study by Brenner et al. (2016) for all member states of the EU found that long-term unemployment is linked to bad self-perceived health, and that the longer the unemployment the higher the incidence of the perception of bad health [16].

Nevertheless, for example, Tøge et al. (2015), using the Survey on Living Conditions (SLC) from 28 European countries (2008–2011), applied fixed-effects regression models but failed to find an association between both variables [24].

In Spain, the only study linking unemployment length with perceived health after the 2008 crisis is the work carried out by Urbanos and González (2015) with data from the National Spanish Health Survey 2011–2012 [19]. Their results indicate that being unemployed has a detrimental effect on mental and self-perceived health, and that this effect increases the longer the unemployment spell lasts.

Socioeconomic conditions and the decrease in family income are linked to poor health indicators [25, 26]. Aittomäki et al. (2012, 2014) used longitudinal data to analyze how health inequalities are associated with the specifics of the labor market and family income [27, 28]. Poverty and material deprivation are risk factors [29, 30] associated with poor perceived health [31] mental illness [32] both for the general population and for specific groups such as children or the elderly [33–35]. In Spain, the available evidence points at material deprivation as a risk factor for health [36, 37].

The international literature has looked into the matter of social public expenditure and health-care expenditure and their impact on the health of individuals, and has found that they have a positive effect across countries both at the global [38, 39], OECD [40, 41], and European levels [1, 42–47]. Conversely, Huijts et al. (2014) found that expenditure on active employment policies, unemployment benefits, and even total social expenditure had a very limited effect (even negative for women) in moderating the detrimental effects of unemployment on self-perceived health [48].

The main goal of the present study is to analyze the relationship between self-perceived health and two dimensions which are intimately linked with changes happened in the Spanish labor market after the financial crisis: long-term unemployment and social deprivation. In addition, a second goal at the regional level is to look into the relationship between regional social public expenditure and self-perceived health. The first goal requires considering four variables of interest: long- and very-long-term unemployment, income, and severe material deprivation. For the second goal two variables will be considered: per capita expenditure on fundamental public services and public per capita health-care expenditure. To this end, a longitudinal database was built using the Spanish SLC 2007–2011. Multilevel methods have been employed to integrate regional variables in order to produce a coherent hierarchy of data.

To the extent of our knowledge, no previous studies have dealt with the relationship between the financial crisis and the perceived health of the population using a set of variables that allows for the simultaneous consideration of long- and very-long-term unemployment, poverty and public social expenditure.

## Methods

With the goal of observing the employment history of given individuals, a database was built from the longitudinal data files of the SLC between 2007 and 2010 and 2008–2011 [49]. Only individuals with continuous presence in the data during the four years were included. Individuals under 16 and over 65 were excluded. The longitudinal database includes 36,420 observations from 9105 individuals in 17 regions during the financial crisis of 2007–2011 in Spain. In the Spanish SLC, perceived health is a categorical variable with five possible answers (very good, good, fair, bad, and very bad), which in most models described in this study are collapsed into two (very good, good: good; fair, bad or very bad: bad).

Table 1 displays the individual and regional variables selected for each level.

**Table 1:** Individual and regional variables used to analyze the relationship between long-term unemployment and health

LEVEL 1 (year): 36,420 observations		
Years	2007–2011	
LEVEL 2 (individual) 9105 individuals		
Dependent variable	Self-perceived health (collapsed): Good (Very good/Good) - Bad (Fair/Bad/Very bad) Self-perceived health: Very good/Good/Fair/Bad/Very bad	
Independent variables		
Individual	Sociodemographic	Age (range: 16–65)^a^ Gender (male/female) Chronic illness or chronic disability (Yes/No)
	Socioeconomic	Education level (Primary/Secondary/Higher) Activity status (Employed/Student/Homemaker/Other inactivity/Unemployed <12 months/ Long-term Unemployed, 12–23 months/Very-long-term unemployed (24–48 months) Equivalent household income^b^ (Neperian logarithm) Severe material deprivation (Yes/No)
LEVEL 3 (regional) 17 regions.		
Regional	Public expenditure policies	Essential public services per capita^b^,^c^ Health-care public expenditure per capita^b^

^a^Centered continuous variable

^b^Nominal values were converted to real values using 2007 as the base year and Consumer Price Index (National Statistics Institute) as a deflator

^c^Essential public services include: health care (primary, specialized, and hospital assistance, public health, clinical research); education (kindergarten, primary, secondary, post-secondary, and higher, grants, support services to education); and social protection (retirement, illness, disability, advanced age, protection of families, unemployment, housing, attention to social exclusion)

Source: the authors

The dependent variable was self-perceived health, as recorded in the SLC under “General health status”. Self-perceived health provides a multidimensional approach to health [50–52]; and is a good predictor of mortality [53, 54], morbidity [55], disability, and use of health services [56–58]. For the purposes of our research, self-perceived health was collapsed as a dichotomous variable: good self-perceived health (very good or good) and bad self-perceived health (fair, bad, or very bad). This dichotomization follows the trend of most of the related literature [59–61], which allowed us to compare results against those of previous research.

Independent variables at the individual level include gender, activity status and education level, in accordance with previous studies on self-perceived health [18, 60–63]. The variable “activity status” combines the answer categories as defined by the subject and the information provided by the question regarding monthly activity (employed, student, homemaker and/or caretaker, inactive (retired, disabled, and other forms of economic inactivity).

Unemployment variable has been categorized in being unemployed less than 12 months, being unemployed between 12 and 23 months, and being unemployed between 24 and 48 months. These categories correspond to what the literature refers to as long-term unemployment (between 12 and 23 months) and very-long-term unemployment (more than 24 months, −between 24 and 48 months-) [16].

Chronic disease may affect the odds of being unemployed and, in turn, a given individual may see their chronic condition worsen due to their losing their job or spending a long time unemployed. In order to check for robustness, our models have been tested with and without this variable [19].

Income level is one of the main components of the social gradient of health [64, 65]. Given the evidence about the moderating role of family income on the link between employment status and individual health, we have introduced the independent variable “equivalent household income” [66]. This variable has been calculated by applying the OECD modified equivalence scale to available household income [67]. The variable “severe material deprivation” was introduced in the model because it is one of the components of the AROPE index (At Risk Of Poverty or social Exclusion). This indicator is obtained from the SLC and is harmonized at the European level: it includes people who declare being unable to afford at least four of a list of nine concepts listed in the Europe 2020 strategy, and who are therefore considered to be at risk of poverty [68].

Regionally, certain ecological variables have been introduced to account for public expenditure policies: expenditure on essential public services (education, health-care, and social protection [69], and public health-care expenditure [70].

Given that our study looks into the relationship between individual and regional variables and perceived health in a simultaneous way, we have employed a random group effects in the constant longitudinal multilevel logistic model. This approach is well suited for hyerarchical structures incorporating different levels of information, in which individuals share certain characteristics due to their belonging to the same higher level (the region), and repeated measurements are available over a certain time span, as it allows for the estimation of variance for each level.

In order to be able to contrast how health is related to long and very long unemployment, income and individual social deprivation, as well as its association with the regional social and economic context, in the present work we have estimated a series of longitudinal multilevel logistic models (level 1: year; level 2: individual; and level 3: region), with random intercept. These multilevel models address the lack of independence of ordinary least squares models through the inclusion of hyerarchical data, and avoid the ecological fallacy (in which aggregated data are interpreted at the individual level) and the atomistic fallacy (in which individual data are interpreted at the aggregated level) [71].

The multilevel logistic regression model points at the dependent variable Y_ijk_ (perceived health; collapsed into good or bad health for year i) following a binomial distribution *Y*_*ijk*_ *~ Binomial(1,π*_*ijk*_*)* with variance Y, conditioned on *π,* Var(*Y*_*ijk*_|*π*_*ijk*_) = (1-*π*_*ijk*_), where *π*_*ijk*_ is the likelihood of presenting the feature of interest for year *i*, being *i* = 2007, …, 2011, *j* the subject, *j* = 1, ..., 9105, and being *k* the region, with *k* = 1, …, 17.

Analytically:

$$ logit\left({y}_{ijk}\right)={\beta}_0+{\sum}_{h=1}^H{\beta}_h{X}_{hijk}+{\sum}_{m=1}^M{\alpha}_m{Z}_{mik}+{\nu}_{0k}+{\mu}_{0 jk}+{\epsilon}_{ijk} $$

where *β*_0_ is the independent term, X_ijk_ the explanatory variables at individual level *j*, and *β*_*h*_its associated coefficients; Z_jk_ are the explanatory variables at the regional level *k*, and *α*_*m*_ its associated coefficients. The error term divides the dependent variable into three parts, once for each hierarchical level.

In addition, and in order to confirm that the loss of information resulting from collapsing perceived health into fewer categories does not skew the results of the estimated odds ratios of the variables of interest, an ordered logit model was estimated with the self-perceived health in its original five categories. This longitudinal multilevel ordered logit model can be written in terms of a latent response y*_ijk_:

$$ {y}_{ijk}^{\ast }={\beta}_0+{\sum}_{h=1}^H{\beta}_h{X}_{hijk}+{\sum}_{m=1}^M{\alpha}_m{Z}_{mik}+{\nu}_{0k}+{\mu}_{0 jk}+{\epsilon}_{ijk} $$

The ordinal of self-assessed health variable *y*_*ijk*_ is related to the latent response via the threshold model: *y*_*ijk*_ = 1 if y*_ijk_ ≤ *k*_*1*_, *y*_*ijk*_ = 2 if *k*_*1*_ < y*_ijk_ ≤ *k*_*2*_, *y*_*ijk*_ = 3 if *k*_*2*_ < y*_ijk_ ≤ *k*_*3*_, *y*_*ijk*_ = 4 if *k*_*3*_ < y*_ijk_ ≤ *k*_*4*_ and *y*_*ijk*_ = 5 if *k*_*4*_ < y*_ijk_ where *k* parameters are the cutpoints, which will be estimated together with parameters *β* and *α* in the model.

In order to be able to estimate the extent to which the areas under analysis (regions) determine individual differences in health status, we calculate the variance partition coefficient (VPC) [72], and the median odds ratio (MOR) of the region as per the latent-variable method [73].

In total, 9 models were developed to estimate the relationship between the variables of interest and self-perceived health in Spain (2007–2011). Starting from the base model, the first three models treat chronic illness differently and use different subsamples (Table 3). Being Model 1 the base model, Model 2 controls for chronic illness, Model 3 excludes from the sample those individuals whose chronic illness appeared during the four follow-up years. Model 4 excludes those individuals who were unemployed at the beginning of the panel (in January 2007 for panel 2007–2010 and in January 2008 for panel 2008–2011), in order to avoid merging recently unemployed with long term unemployed people. By estimating this model we may check our results for robustness regarding the presence of individuals who were already unemployed at the beginning of the panel.

The following four models reproduce the previous sequence, but with a subsample including only those individuals who reported having good or very good health at the begining of the panel (Table 4). In other words, in these models none of the individuals who later found themselves unemployed, particularly for the long or very long term, reported to perceive their health as poor.

The goal of these models is testing our coefficients for sensitivity when good-health individuals are selected at the beginning of their panel. Some of them may fall ill and become unemployed for this reason, but among the unemployed the percentage of individuals reporting good health has increased (from 78.56% in 2007 to 82.44% in 2011; see Table 2). The last model, number 9, estimates an longitudinal, multilevel, ordered logit model in order to assess the extent to which the results found for the variables of interest are affected by the loss of information caused by collapsing self-perceived health from its five original categories into just two.

**Table 2:** Self-perceived health for population subsets, Spain 2007–2011

Collapsed Health	Bad Health %	Good Health %	Total
LEVEL 1: YEARS			
2007	78.56% (*N* = 3521)	21.44% (*N* = 961)	100% (*N* = 4482)
2008	81.51% (*N* = 7311)	18.49% (*N* = 1658)	100% (*N* = 8969)
2009	78.93% (*N* = 7102)	21.07% (*N* = 1896)	100% (*N* = 8998)
2010	79.39% (*N* = 7131)	20.61% (*N* = 1851)	100% (*N* = 8982)
2011	82.44% (*N* = 3705)	17.56% (*N* = 789)	100% (*N* = 4494)
Unemployed 2007	71.47% (*N* = 228)	28.53% (*N* = 91)	100% (*N* = 319)
Unemployed 2008	73.96% (*N* = 531)	26.04% (*N* = 187)	100% (*N* = 718)
Unemployed 2009	75.49% (*N* = 878)	24.51% (*N* = 285)	100% (*N* = 1163)
Unemployed 2010	77% (*N* = 974)	23% (*N* = 291)	100% (*N* = 1265)
Unemployed 2011	82.22% (*N* = 555)	17.78% (*N* = 120)	100% (*N* = 675)
LEVEL 2: INDIVIDUAL			
Sex			
Male	81.84% (*N* = 14,147)	18.16% (*N* = 3139)	100% (*N* = 17,286)
Female	78.45% (*N* =14,623)	21.55% (*N* = 4016)	100% (N = 18,639)
Age			
> 25 years	94.84% (*N* = 5108)	5.16% (*N* = 278)	100% (*N* = 5386)
25–34	91.65% (*N* = 5938)	8.35% (*N* = 541)	100% (*N* = 6479)
35–44	82.78% (N = 7131)	17.22% (*N* = 1483)	100% (*N* = 8614)
45–54	73.51% (*N* = 6714)	26.49% (*N* = 242)	100% (*N* = 9134)
55–65	61.45% (*N* = 3879)	38.55% (*N* = 2433)	100% (*N* = 6312)
Education level			
Primary	63.81% (*N* = 4207)	36.19% (*N* = 2386)	100% (*N* = 6593)
Secondary	82.43% (*N* = 15,329)	17.57% (*N* = 3267)	100% (N = 18,596)
Higher	89.26% (*N* = 8905)	10.74% (*N* = 1071)	100% (*N* = 9976)
Employment Status			
Employed	85.39% (N = 18,571)	14.61% (*N* = 3177)	100% (*N* = 21,748)
Unemployed	76.47% (*N* = 3166)	23.53% (N = 974)	100% (*N* = 4140)
Student	96.44% (*N* = 3111)	3.56% (*N* = 115)	100% (*N* = 3226)
Homemaking	66.38% (*N* = 2654)	33.62% (*N* = 1344)	100% (*N* = 3998)
Other inactive	45.03% (N = 1265)	54.97% (*N* = 1544)	100% (*N* = 2809)
Unemployed <12 months	85.73% (*N* = 3455)	14.27% (*N* = 575)	100% (*N* = 4030)
Unemployed 13–23 months	76.91% (*N* = 3300)	23.09% (*N* = 991)	100% (*N* = 4291)
Unemployed 24–48 months	72.21% (*N* = 2009)	27.84% (*N* = 775)	100% (*N* = 2784)
Equivalent household income			
(−49,189.42, -29,331.98€)	33.33% (*N* = 2)	66.67% (N = 4)	100% (N = 6)
-9474.53€	69.23% (*N* =36)	30.77% (N = 16)	100% (*N* = 52)
10,382.91€	74.21% (*N* = 8697)	25.79% (*N* = 3023)	100% (*N* = 1172)
30,240.35€	82.39% (*N* =18,036)	17.61% (*N* = 3855)	100% (N = 21,891)
50,097.79€	88.34% (*N* =1781)	11.66% (*N* = 235)	100% (*N* = 2016)
69,955.23€	90.34% (*N* = 187)	9.66% (N = 20)	100% (*N* = 207)
89,812.67€	92.31% (*N* =24)	7.69% (N = 2)	100% (N = 26)
129,527.60€	100% (*N* = 4)	0% (*N* = 0)	100% (N = 4)
149,385.00€	100% (N =3)	0% (N = 0)	100% (N = 3)
Severe material deprivation	58.95% (*N* = 484)	41.05% (*N* = 337)	100% (*N* = 821)
LEVEL 3:REGIONS			
Galicia	71.08% (*N* = 1870)	28.92% (*N* = 761)	100% (*N* = 2631)
Asturias	82.8% (*N* = 1386)	17.2% (*N* = 288)	100% (*N* = 1674)
Cantabria	81% (*N* = 942)	19% (*N* = 221)	100% (N = 1163)
Pais vasco	85.5% (*N* = 1533)	14.5% (*N* = 260)	100% (*N* = 1793)
Navarra	87.14% (*N* = 1226)	12.86% (*N* = 181)	100% (*N* = 1407)
Rioja	82.4% (*N* = 1072)	17.6% (*N* = 229)	100% (*N* = 1301)
Aragón	80.58% (*N* = 1324)	19.42% (N = 319)	100% (*N* = 1643)
Madrid	82.27% (*N* = 2515)	17.73% (*N* = 542)	100% (*N* = 3057)
Castilla y León	77.78% (*N* = 1869)	22.22% (*N* = 534)	100% (*N* = 2403)
Castilla la Mancha	81.47% (*N* = 1609)	18.53% (*N* = 366)	100% (*N* = 1975)
Extremadura	82.2% (*N* = 1288)	17.8% (*N* = 279)	100% (*N* = 1567)
Cataluña	81.24% (*N* = 2846)	18.76% (*N* = 657)	100% (*N* = 3503)
Comunidad Valenciana	80.9% (*N* = 2393)	19.1% (*N* = 565)	100% (*N* = 2958)
Baleares	83.86% (*N* = 904)	16.14% (*N* = 174)	100% (*N* = 1078)
Andalucía	78.01% (*N* = 3359)	21.99% (*N* = 947)	100% (*N* = 4306)
Murcia	79.31% (*N* = 1296)	20.69% (*N* = 338)	100% (*N* = 1634)
Canarias	73.03% (*N* = 1338)	26.97% (*N* = 494)	100% (*N* = 1832)

Source: Prepared by the authors based on data from the Survey on Living Conditions. Instituto Nacional de Estadística (2014) http://www.ine.es/dyngs/INEbase/es/operacion.htm?c=Estadistica_C&cid=1254736176807&menu=ultiDatos&idp=1254735976608. Accessed 5 Jan 5 2017

All models of multilevel regression were planned and executed using the STATA 14.0 statistical software package [74].

## Results

Table 2 shows the data regarding self-perceived health according to individual, family, and regional variables. The variable of interest at the individual level (activity status) shows that inactive (42.4%), homemaking (33.6%), or unemployed individuals (23.5%) report having worse health than those who are employed (14.6%) or studying (3.6%). Among the unemployed, the time spent with no employment affects how one’s health is perceived: the proportion of the unemployed who report good health when being unemployed for less than 12 months is higher (85.7%) than for those who have been unemployed for between one and two years (76.9%). This percentage is lowest among those who have been unemployed for between two and four years (72.16%).

As for the rest of individual variables, the analysis points at males having better self-perception of health (81.8%) than females (78.4%). Education level is associated with improved perception of one’s health, and age is linked with worsened perception of one’s health. As family income level increases, so does reported health, and being a member of a severely materially deprived household has a strong negative relationship with subjective health.

Table 3 shows the results of the first four multilevel models, which calculates the modulating effect of individual and regional variables on the association between unemployment and self-perceived health, depending on whether chronic illness is being controlled for or not (Models 1 and 2) or by dropping all the chronically ill people from the sample as in Model 3. As it has been described in the methodology section, in model 4 unemployed individuals at the beginning of panel are dropped.

**Table 3:** Multilevel model of the association of individual and regional variables with self-perceived health, Spain 2007–2011

SEQUENTIAL ESTIMATION OF MODELS 2007–2011 PANEL	Model 1 PURE PANEL ORIGINAL DATABASE Without controlling for chronically ill	Model 2 PURE PANEL ORIGINAL DATABASE	Model 3 NO CHRONICALLY ILL INDIVIDUALS ALTOGETHER	Model 4 EXCLUDING UNEMPLOYED AT THE BEGINNING OF THE PANEL
SAMPLE	34,692 OBSERVATIONS 9003 INDIVIDUALS	34,692 OBSERVATIONS 9003 INDIVIDUALS	27,008 OBSERVATIONS 8306 INDIVIDUALS	32,324 OBSERVATIONS 8385 INDIVIDUALS
INDIVIDUAL LEVEL: YEARS 2007–2011				
Chronic illness	–	23.54 (21.07–26.3) ***	–	23.24 (20.69–26.12) ***
Centered age	1.09 (1.09–1.1)***	1.06 (1.06–1.07) ***	1.07 (1.06–1.08) ***	1.06 (1.06–1.07) ***
Female	1.37 (1.18–1.59) ***	1.42 (1.25–1.62) ***	1.53 (1.3–1.8) ***	1.41 (1.23–1.62) ***
Education level (primary as reference)				
Secondary education	0.62 (0.53–0.73) ***	0.63 (0.54–0.73) ***	0.70 (0.58–0.84) ***	0.62 (0.53–0.72) ***
Higher education	0.28 (0.23–0.34) ***	0.31 (0.26–0.37) ***	0.32 (0.26–0.41) ***	0.31 (0.26–0.38) ***
Activity (employed as reference)				
Student	0.62 (0.44–0.88) ***	0.49 (0.36–0.67) ***	0.34 (0.21–0.54) ***	0.51 (0.37–0.71) ***
Homemaking	1.46 (1.21–1.76) ***	1.23 (1.03–1.46) ***	0.99 (0.79–1.23) ***	1.28 (1.07–1.54) ***
Other inactive	4.84 (4.-5.85)***	3.07 (2.57–3.67) ***	1.96 (1.51–2.53) ***	3.13 (2.6–3.78) ***
Long and Very long Unemployment				
Unemployed between 1 and 11 months	1.08 (0.85–1.38)	0.96 (0.78–1.18)	0.99 (0.76–1.28)	0.99 (0.8–1.24) ***
Unemployed between 12 and 23 months	1.61 (1.29–2.01) ***	1.41 (1.17–1.7) ***	1.37 (1.08–1.73) ***	1.43 (1.16–1.76) ***
Unemployed between 24 and 48 months	2.32 (1.79–3.02) ***	1.81 (1.46–2.26) ***	1.72 (1.31–2.26) ***	1.60 (1.19–2.13) ***
Socioeconomic variables				
Equivalent household income	0.80 (0.74–0.86) ***	0.75 (0.69–0.8) ***	0.76 (0.7–0.83) ***	0.72 (0.67–0.78) ***
Severe material deprivation	2.10 (1.57–2.8) ***	1.95 (1.46–2.6) ***	1.74 (1.19–2.55) ***	1.97 (1.43–2.72) ***
REGIONAL LEVEL: 17 REGIONS				
Health-care expenditure	1.00 (1.00–1.001)***	0.99 (0.99–1.001)	1.00 (0.99–1.001)	1.00 (0.99–1.001)
Fundamental public services	0.99 (0.99–0.99)***	0.99 (0.99–0.99)***	0.99 (0.99–0.99)***	0.99 (0.99–0.99)***

*p*-value < 0.01 = ***; *p*-value< 0.05 = **; *p*-value < 0.10 = *. Source: Prepared by the authors based on data from the Survey on Living Conditions. Instituto Nacional de Estadística (2014). http://www.ine.es/dyngs/INEbase/es/operacion.htm?c=Estadistica_C&cid=1254736176807&menu=ultiDatos&idp=1254735976608. Accessed 27 Dec 2016

Table 4 shows the results of the same sequence of models when the subsample contains those individuals who reported having good or very good health at the beginning of the panel. It also shows the results of the longitudinal, multilevel, ordered logit model.

**Table 4:** Multilevel model of the association of individual and regional variables with self-perceived health for people in good health at the beginning of the panel, Spain 2007–2011

SEQUENTIAL ESTIMATION OF MODELS 2007–2011 PANEL Excluding bad health at the beginning of the panel	Modelo 5 PURE PANEL Not controlling for chronically ill individuals	Model 6 PURE PANEL ORIGINAL DATABASE	Model 7 NO CHRONICALLY ILL INDIVIDUALS ALTOGETHER	Model 8 WITHOUT UNEMPLOYED AT THE BEGINNING OF THE PANEL	Model 9 PURE PANEL ORIGINAL DATABASE SELF-PERCEIVED HEALTH IN 5 CATEGORIES ORDERED MEOLOGIT
SAMPLE	28,142 OBSERVATIONS 7313 INDIVIDUALS	28,142 OBSERVATIONS 7313 INDIVIDUALS	24,321 OBSERVATIONS 7140 INDIVIDUALS	26,372 OBSERVATIONS 8218 INDIVIDUALS	34,692 OBSERVATIONS 9003 INDIVIDUALS
Chronic illness	–	19.86 (17.-23.19) ***	40.21 (34.89–46.34) ***	–	19.76 (16.8–23.24) ***
Centered age	1.06 (1.05–1.07) ***	1.04 (1.03–1.05) ***	1.06 (1.05–1.07) ***	1.05 (1.04–1.06) ***	1.04 (1.04–1.05) ***
Female	1.29 (1.08–1.54) ***	1.32 (1.12–1.55) ***	1.50 (1.3–1.74) ***	1.34 (1.08–1.66) ***	1.32 (1.12–1.57) ***
Secondary education	0.69 (0.56–0.86) ***	0.65 (0.53–0.79) ***	0.60 (0.51–0.71) ***	0.69 (0.54–0.89) ***	0.65 (0.53–0.8) ***
Higher education	0.38 (0.3–0.5) ***	0.37 (0.29–0.47) ***	0.27 (0.22–0.33) ***	0.35 (0.25–0.48) ***	0.37 (0.29–0.48) ***
Student	0.57 (0.38–0.86) ***	0.49 (0.33–0.73) ***	0.48 (0.33–0.7) ***	0.37 (0.21–0.66) ***	0.51 (0.34–0.77) ***
Homemaking	1.08 (0.84–1.38)	1.02 (0.8–1.3)	1.23 (1.01–1.5) **	0.90 (0.65–1.24)	1.01 (0.78–1.3)
Other inactive	2.26 (1.71–3.00) ***	1.67 (1.26–2.2) ***	3.31 (2.69–4.08) ***	1.49 (1.-2.22) ***	1.65 (1.24–2.21) ***
Unemployed between 1 and 11 months	1.10 (0.83–1.46)	0.97 (0.75–1.25)	0.90 (0.71–1.15)	0.99 (0.71–1.4)	1.02 (0.77–1.33) ***
Unemployed between 12 and 23 months	1.67 (1.29–2.17) ***	1.52 (1.2–1.93) ***	1.45 (1.18–1.8) ***	1.38 (1.02–1.88) ***	1.56 (1.2–2.03) ***
Unemployed between 24 and 48 months	1.93 (1.42–2.63) ***	1.64 (1.23–2.17) ***	1.84 (1.37–2.46) ***	1.54 (1.07–2.21) ***	1.71 (1.2–2.43) ***
Equivalent household income	0.81 (0.74–0.89) ***	0.77 (0.7–0.84) ***	0.74 (0.68–0.8) ***	0.76 (0.67–0.85) ***	0.76 (0.69–0.84) ***
Severe material deprivation	2.40 (1.62–3.54) ***	2.13 (1.44–3.17) ***	2.29 (1.65–3.18) ***	2.01 (1.2–3.37) ***	2.11 (1.36–3.29) ***
Health-care expenditure	1.00 (0.99–1.00)	1.00 (0.99–1.00)	1.00 (1.00–1.00)*	1.00 (1.00–1.00)*	1.00 (1.00–1.00)
Fundamental public services	1.00 (1.00–1.00)***	1.00 (1.00–1.00) ***	1.00 (1.00–1.00) ***	1.00 (1.00–1.00) ***	1.00 (1.00–1.00) ***

*p*-value < 0.01 = ***; *p*-value < 0.05 = **; *p*-value < 0.10 = *. Ref. Reference cathegory. Source: Prepared by the authors based on data from the Survey on Living Conditions. Instituto Nacional de Estadística (2014). http://www.ine.es/dyngs/INEbase/es/operacion.htm?c=Estadistica_C&cid=1254736176807&menu=ultiDatos&idp=1254735976608. Accesed 27 Dec 2016

**Fig. 1:**
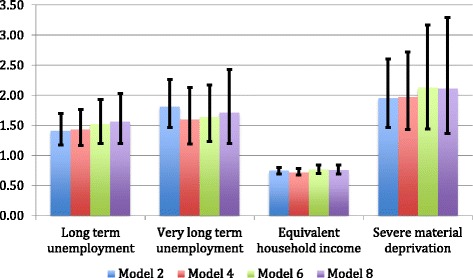
Odds ratios for the association between long and very-long term unemployment, income, job insecurity, poverty, and self-perceived health in Spain (2007–2011). Source: Prepared by the authors based on data from the Survey on Living Conditions. Instituto Nacional de Estadística (2014). http://www.ine.es/dyngs/INEbase/es/operacion.htm?c=Estadistica_C&cid=1254736176807&menu=ultiDatos&idp=1254735976608. Accessed 27 Dec 2016

The multilevel models used to estimate the association of long- and very-long-duration unemployment with the self-perceived health of individuals between 2007 and 2011 shows that long- and very–long-duration unemployment are associated with how health is subjectively perceived. Figure 1 shows the odds ratios for long- and very-long duration unemployment and other variables of interest in the main models.

By comparing the results of the first three estimations, it is apparent that there are no significant changes between the odds ratios of the variables of interest (Table 3). For instance, and regarding the long-term unemploment (between 12 and 23 months) and very-long-term unemployment (between 24 and 48 months) variables, odds ratios for models 1, 2, and 3 are 1.61, 1.41, and 1.37 for long-term unemployment, and 2.32, 1.81 and 1.72 for very-long-term unemployment. To sum up, after excluding chronically ill subjects from the sample the model remains stable regarding the relationship between long-term and very-long-term unemployment, income, poverty, and poor perceived health.

In addition, model 4 exclude subjects who were unemployed at the beginning of the panel. The odds ratios for these models are also similar to those of the full sample concerning the three individual variables of interest and the two regional variables (Table 3). For instance, for the variables concerning long-term and very-long-term unemployment odds ratios are 1.43 and 1.60. The analysis of the subsample that included only those individuals reporting good or very good at the beginning of the panel shows that results remain stable (Table 4, Fig. 1). In Model 8, in which subjects reported good health, and were not unemployed at the beginning of the panel, the odds ratio for the long-term unemployed (between 12 and 23 months) is 1.56, which increases for the very-long-term unemployed (between 24 and 48 months), 1.71.

The estimation performed with the longitudinal ordered logit model (Model 9) yields similar odds ratios in the variables of interest to those of the base model. For instance, concerning long-term and very-long-term unemployment, coefficients are 1.41 and 1.81 in Model 2, and 1.22 and 1.60 in the longitudinal ordered model. These results hold for all other variables of interest across all estimations.

In basic models, and controlling for chronic illness (numbers 2 and 6), the household income variable was associated with a reduction of the odds of declaring bad health of 25% and 23% respectively for each additional percentage point in income. Being a member of a household with severe material deprivation increased by 95% and 113% respectively the odds of perceiving one’s health as bad compared with households not presenting severe material deprivation. In Tables 3 and 4 and the Fig. 1, these data are consistent across the models. This is the case of Model 4, which excluded the unemployed at the beginning of the panel (28% increase for income and 97% decrease for severe material deprivation) and of the model dealing only with individuals in good or very good health, excluding the unemployed at the beginning of the panel (24% increase for income and 111% decrease for severe material deprivation, Model 8). The rest of individual variables behaved according to what has been described in the previous literature.

The analysis of VPC in the basic model number 2 showed that 1.4% of variance in the odds of reporting bad health can be attributed to the modulating effect that regional variables exert on the association between unemployment and self-perceived health. The calculation of the MOR shows that, when comparing two randomly selected regions, the likelihood of declaring bad health was 34% higher in one than in the other (in the median case).

Results regarding the influence of regional public expenditure on the association between long- and very-long-term unemployment and self-perception of health show that expenditure on essential public services is associated with better self perceived health: for each additional percentage point of increase in health-care, education, and social protection the odds of declaring bad health decreases by 0.01% (in every model, 1–9). Public health-care expenditure per capita did not yield statistically significant results.

## Discussion

Before discussing our results, some limitations must be acknowledged. Firstly, and given the bidirectional nature of the relationship between the variables of interest and perceived health, this study is not able to establish a causal relationship between the associations we have identified [75]. This becomes particularly relevant regarding the link between bad perceived health and long-term unemployment.

The literature has identified two processes linking bad perceived health and unemployment. On the one hand, the causal hypothesis suggests that unemployment is a risk factor for health. On the other, the selection hypothesis states that it is poor health which excludes workers from the labor market [76–78]. Three metaanalyses concluded that longitudinal studies provide enough evidence for both the causal and the selection hypotheses [79–81]. More recently, some studies have yielded certain evidence supporting the latter [82, 83]. The work of Reeves et al. (2014) suggests that the financial crisis in Europe has had particularly severe effects on people with bad health, who are more prone to losing their jobs when market conditions worsen [84]. Heggebø and Dahl (2015), however, pointed out that while the selection effect has remained constant throughout time in the EU, in countries like Spain, where the financial crisis has brought about a swift increase in unemployment and high rates of unemployment population, a change has taken place in the breakdown of the unemployed population, which now includes a higher percentage of individuals who report to have good health [85]. This overrepresentation of the healthy among the unemployed can be interpreted as a consequence of a massive, sudden loss of employment, and supports the causal hypothesis. During the first years after the onset of the crisis (which is the period covered in our analysis), the destruction of employment affected temporary workers on a greater measure, since their lay-off costs are smaller than those of permanent workers [10].

Our results are consistent with this hypothesis. The percentage of unemployed individuals reporting good health has increased from 78.56% in 2007 to 82.44% in 2011. In addition, when only considering the subsample reporting good health at the beginning of the study, results show a robust association between long-term unemployment and bad perceived health, which increases with the time spent unemployed.

However, the selection effect may well play an important role in long-term unemployment, particularly when employers use poor health as an indicator for low productivity in their recruitment processes, in a context of low labor demand brought about by the financial crisis [84]. Subsequent studies should explore the evolution of unemployment since the end of the financial crisis (2014), in a context of sustained creation of jobs, and contemplate in their methodological approaches the need to analyze the endogenic nature of the association between unemployment and health, for example by using structural equation modeling [27].

Secondly, although self-perceived health is one of the best global health indicators, several significant dissonances have been described with objective indicators of morbimortality when populations have been compared [86, 87]. Amartya Sen (2009) suggested employing a social context to examine the statistics on the perception of bad health, with a critical analysis of positional perspectives [88]. Contemplating some features of the labor market which may amount to risk factors for health, like job insecurity or involuntary part-time work, might provide a more thorough and detailed analysis of labor markets and their influence on health.

Yet another limitation originates from the SLC not including individual lifestyle information. In this regard it only records data concerning self-perceived health, chronic illness, and limitations for activity in daily life. This is however the only survey conducted in Spain to offer longitudinal information about the activity and employment status of individuals.

The present study offers evidence of the association between long- and very-long-term unemployment, loss of family income, and living in a household that is severely materially deprived with bad self-perception of health. All estimated models show similar and consistent results for all variables of interest.

According to our results, bad perceived health is associated with long- and very-long-term unemployment, and worsens as the time spent unemployed increases. This is in agreement with part of the literature published in this regard before the onset of the financial crisis [16–18, 22, 23] and with the work of Urbanos and González (2015), regarding the Spanish situation after the crisis [19].

Some evidence exists that certain health conditions and causes of mortality (such as suicide) increase due to the deleterious effect of recessions on mental health [2, 5]. Ähs and Westerling (2005) compared self-perceived bad health during times of low (1983–1989) and high (1992–1997) rates of unemployment in Sweden and, after controlling for sociodemographic factors and long-term health conditions, differences in self-perceived health between the employed and the unemployed were higher at times of high unemployment [89]. Drydakis (2015) recently published his results regarding the negative impact of unemployment on the mental health and self-perceived health of Greek individuals in the period 2008–2013 [90].

However, our study revealed that, by following the professional history of individuals along four years since the onset of the crisis, a robust association appears between long- and very-long-term unemployment and the deterioration of the perception of their own health, after controlling for other individual and regional variables.

Our results also show that, after only one year of unemployment, perceived health worsens. One tentative explanation for this phenomenon is that a change in expectations takes place when the reality of being unemployed and losing income settles in and reveal itself as a permanent situation, thus increasing uncertainty about the future and causing stress and anxiety [91].

Labor policies aimed at reducing the long-term unemployment rate as a strategy to improve the health of the population are particularly attractive, since they are synergistic with macroeconomic policies of fiscal consolidation and sustained economic growth [92]. In a recent research, Doménech and González Páramo (2017) have shown how a reduction of 8% in structural unemployment would in the long term mean an increase in GDP and public expenditure per working-age population of more than 20% [93]. Additionally, according to the results of this study, health would likely be improved by the reduction of long-term unemployment and the reduction of social deprivation.

In the present study household income decreased the odds of reporting bad health by 16% to 28% (depending on the model) for each percentage point of income increase. Conversely, being member of a household with severe material deprivation affected the perception of health and increased the odds of perceiving one’s health as bad by 70% to 140% (depending on the model). These results are in agreement with several others that found a positive correlation between unemployment, low social and economic level, and bad health [25, 28, 78, 94–97]. In Spain, the link between material deprivation and bad health was already proved in studies performed both prior to the onset of the financial crisis [36] and after [37].

The fact that severe material deprivation is associated with bad health is probably due to two mechanisms: an increase in the general susceptibility to illness and a set of specific factors, which increase the risk of death (healthy lifestyle, overweight, obesity, alcohol consumption, smoking, etc. [98]. The work of Ayllón and Gábos (2016) suggests that a vicious circle is established in which living in conditions of material deprivation for a long time erodes the human and social capital of individuals and worsens their health [99]. Long-term unemployment thus breeds poverty and material deprivation, which in turn decrease the chances of entering the labor market.

Regional per capita expenditure on essential public services is associated with better perceived health, although its influence is limited, whereas per capita expenditure on health-care did not show to have any significant relationship with self-perceived health. These results do not agree with those of other authors [40, 41, 100]. For instance, Ng and Muntaner (2015) found that expenditure on health-care, social services, and education reduced mortality rates in the provinces of Canada [101]. Huijts et al. (2014), on the other hand, did not find a link between social protection policies, health-care expenditure, and perceived health [48].

## Conclusions

To conclude, this is the first longitudinal study carried out in Spain after the financial crisis to analyze the joint association of long-term unemployment, income, poverty, and severe material deprivation (closely derived from long-term unemployment) with bad perceived health. Our results provide robust evidence that long-term unemployment is related to bad health.

Finally, by using multilevel models we were able to find robust estimators regarding the relationship between social and health-care public expenditure policies in the Spanish regions and perceived health, which turned out to be limited in the case of the former and non significant for the latter.

Our results are particularly relevant for the design of public policies aimed at reducing the weight of social determinants in health. Specifically, these results should be considered when formulating active employment policies, safety nets for the long-term unemployed, and policies of redistribution focused on families with low-income levels and material deprivation.

## Abbreviations

AROPE: At Risk Of Poverty or social Exclusion
MOR: Median Odds Ratio
SLC: Survey on Living Conditions
VPC: Variance Partition Coefficient
